# Analysis of Immune Escape Variants from Antibody-Based Therapeutics against COVID-19: A Systematic Review

**DOI:** 10.3390/ijms23010029

**Published:** 2021-12-21

**Authors:** Daniele Focosi, Fabrizio Maggi, Massimo Franchini, Scott McConnell, Arturo Casadevall

**Affiliations:** 1 North-Western Tuscany Blood Bank, Pisa University Hospital, 56124 Pisa, Italy; 2 Department of Medicine and Surgery, University of Insubria, 21100 Varese, Italy; fabrizio.maggi63@gmail.com; 3 Laboratory of Microbiology, Azienda Socio Sanitaria Territoriale Sette Laghi, 21100 Varese, Italy; 4 Division of Transfusion Medicine, Carlo Poma Hospital, 46100 Mantua, Italy; massimo.franchini@asst-mantova.it; 5 Department of Medicine, Johns Hopkins School of Public Health, Baltimore, MD 21218, USA; smcconn8@jhu.edu (S.M.); acasade1@jhu.edu (A.C.); 6 Department of Medicine, Johns Hopkins School of Medicine, Baltimore, MD 21218, USA

**Keywords:** SARS-CoV-2, COVID-19, convalescent plasma, viral clearance

## Abstract

The accelerated SARS-CoV-2 evolution under selective pressure by massive deployment of neutralizing antibody-based therapeutics is a concern with potentially severe implications for public health. We review here reports of documented immune escape after treatment with monoclonal antibodies and COVID-19-convalescent plasma (CCP). While the former is mainly associated with specific single amino acid mutations at residues within the receptor-binding domain (e.g., E484K/Q, Q493R, and S494P), a few cases of immune evasion after CCP were associated with recurrent deletions within the N-terminal domain of the spike protein (e.g., ΔHV69-70, ΔLGVY141-144 and ΔAL243-244). The continuous genomic monitoring of non-responders is needed to better understand immune escape frequencies and the fitness of emerging variants.

## 1. Introduction

The SARS-CoV-2 spike protein is the target of neutralizing antibody (nAb)-based therapeutics. Control of the COVID-19 pandemic is being hampered by continued evolution of SARS-CoV-2, which includes mutations in the spike protein that can affect immunogenicity and antibody-mediated neutralization. Evolutionary modeling suggests that SARS-CoV-2 strains harboring 1–2 deleterious mutations naturally exist, and their frequency increases steeply under positive selection by monoclonal antibodies (mAb) and vaccines [1]. In 2% of COVID cases, SARS-CoV-2 variants with multiple mutations occur, including in the spike glycoprotein, which can become the dominant strains in as little as one month of persistent in-patient virus replication [2]. While mutations can occur as a natural phenomenon of SARS-CoV-2 RNA replication and editing, the pace of mutagen emergence can also be affected by small-chemical antivirals (e.g., remdesivir [3] or molnupiravir [4]). Since antibody-based therapies targeting the spike protein would also put selective pressure on SARS-CoV-2, it is reasonable to assume that widespread deployment of nAb-based therapeutics could accelerate spike immune escape by selecting for variants that resist neutralization.

Mutations that confer in vitro resistance to therapeutic anti-spike mAbs have been characterized with various methods and are informative about treatment-emergent immune escape. Deep mutational scanning (DMS) predicts protein expression, ACE2 binding, and mAb binding [5]. The method was first deployed with yeast display libraries [6], then evolved to phage display libraries (https://jbloomlab.github.io/SARS-CoV-2-RBD_MAP_clinical_Abs/) [7] and finally mammalian cell surface display [8]. nAb binding is common within the fusion peptide and in the linker region before heptad repeat (HR) region 2. The complete escape maps forecast SARS-CoV-2 mutants emerging during treatment with mAbs and allow the design of escape-resistant nAb cocktails. A complete map of SARS-CoV-2 RBD mutations that escape bamlanivimab and its cocktail with etesevimab has been generated [9,10].

Although DMS was also applied to polyclonal antibodies in COVID-19-convalescent plasma (CCP) [11], the problem is much more complex, such that it is almost impossible to identify escape mutations in CCP or vaccinee-elicited sera, given the huge heterogeneity in antibody response among CCP donors and vaccinees, respectively. In vitro, continuous passaging of SARS-CoV-2 in the presence of a CCP unit with nAb titer >1:10^4^ led to ΔF140 spike mutation at day 45, followed by E484K at day 73, and an insertion in the N-terminal domain (NTD): these accumulating mutations led to complete immune escape [12]. Similarly, K417N, E484K, and N501Y mutations were selected when pseudotyped SARS-CoV-2 was cultured in the presence of vaccine-elicited mAbs [13]. Although some have speculated that the large-scale use of CCP for COVID-19 could have played a role in the emergence of variants, there is no evidence for such an effect and the most likely explanation for the regular emergence of variants has been the huge number of affected individuals since each infection case provides a natural opportunity for variant creation [14].

In vivo, while intrahost-SARS-CoV-2 mutation development is typically very low [15], faster mutation rates (referred to as “accelerated evolution”) have been found in longitudinal studies of immunodeficient patients who had persistent SARS-CoV-2 infections for several months and were treated with nAb-based therapeutics. In this study, we analyze and compare the available mutational data from SARS-CoV-2 under in vitro and in vivo selection and demonstrate that mAb and polyclonal (CCP) therapies elicit different types of mutational patterns.

## 2. Materials and Methods

**Figure 1:**
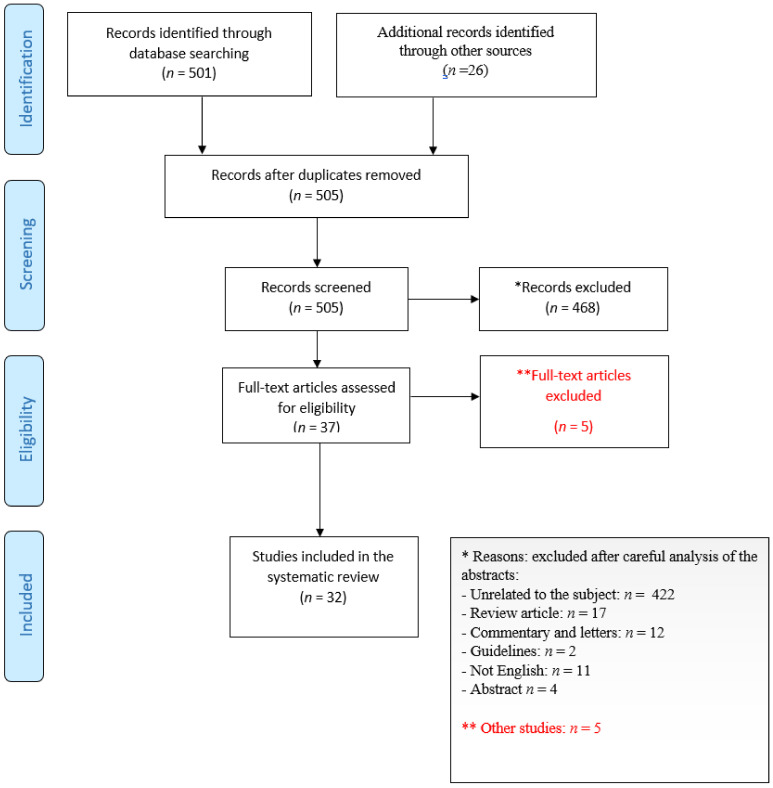
PRISMA flow diagram of study selection.

We mined PubMed (which also indexes the bioRxiv and medrXiv preprint servers) for keywords related to COVID-19 (“COVID-19”, “SARS-CoV-2”), immune escape (“immune escape”, “treatment-emergent resistance”) and nAb-based therapeutics (“convalescent plasma”, “casirivimab”, “imdevimab”, “bamlanivimab”, “etesevimab”, “regdanvimab”) both in vitro and in vivo. Clinical cases were annotated for eventual underlying immune deficiency, concurrent treatments and outcome. Figure 1 reports the study selection process according to PRISMA 2020 guidelines [16].

The 3D structural coordinates of the full spike protein (PDBID 6VXX; residues 27–1252) [16] and the receptor binding domain (PBDID 7BWJ; residues 319–529) [17], solved by cryo-electron microscopy and X-ray crystallography, respectively, were used to map mutational positions of interest. Mapping on the full spike was used to illustrate the diverse set of mutations throughout the spike glycoprotein, while the mutations localized to the RBD were illustrated using the more complete structural model obtained through crystallography. The mutations identified in each condition of in vivo or in vitro selection were tabulated and highlighted on the structures using color coding with PyMOL v.2.4.1. (Schrodinger, Mannheim, Germany) [18].

## 3. Results

Our literature search revealed 32 papers that were then manually inspected to determine whether they included relevant information that was then retrieved, evaluated and organized into Tables.

Table 1 summarizes spike protein mutations associated with in vitro resistance to mAbs targeting this protein. These mutations were used to filter the clinical case reports of treatment resistance for evidence of immune escape (Table 2).

Table 3 summarizes the spike mutations found in clinical cases after CCP treatment, where immune escape can be hypothesized to have occurred based on treatment failure, with the caveat that there is no definitive proof of immune escape due to heterogeneity of the (uncharacterized) polyclonal response.

Table 4 summarizes the data from reports of within-host clonal evolution within immunosuppressed patients not treated with nAb-based therapeutics.

**Figure 2:**
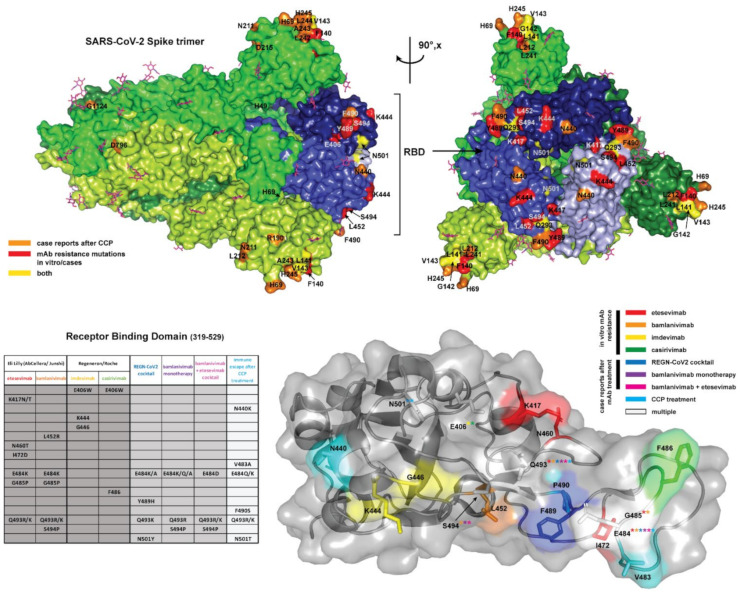
(**Top panel**) The full SARS-CoV-2 S (spike) glycoprotein homotrimer (PDBID 6VXX) [16] in the prefusion conformation is shown in surface representation, with each spike monomer colored a different shade of green. N-linked glycosylations which were resolved in the cryo-EM map in this structure (16/22 sequons per protomer) are displayed as magenta sticks. The receptor binding domains (RBDs), in the closed state, are highlighted in 3 shades of blue corresponding to the shade of the corresponding trimer. Escape mutations from case reports of patients treated with CCP are highlighted in orange. Spike mutations associated with immune escape from clinically approved mAb treatments in vitro or from case reports are highlighted in red, while escape mutations identified in both patients who received clinically approved mAb treatments and CCP treatment are colored yellow. The full spike is shown oriented along the long axis (left) and rotated 90 degrees to display mutations concentrated in the RBDs. Note that mutations located on unresolved loops on the cryo-EM map of the full spike are not visualized (L18, V70, Y144, Y145, D146, R246, W258, G446, N460, I472, V483, E484, G485, F486, R682, N1178 and C1250). (**Bottom panel**) A table summarizing escape mutations localized to the RBD resulting from mAb treatments in vitro and case reports, as well as from CCP treatment. The crystal structure of single RBD domain (PBDID: 7BWJ) [17] from a more complete model (no missing loops) is displayed in surface view with the secondary structure superimposed in cartoon representation. Each escape mutation residue is highlighted by coloration according to the legend to right, and sidechains shown as sticks. In cases where a certain position corresponds to escape mutations from multiple treatments, the position is colored white and the label includes asterisks with the colors corresponding to each treatment where the escape mutation was identified. All figures were generated in PyMOL [18].

Figure 2 depicts the spike RBD mutations of concern for mAb binding detected in vitro and in vivo and the spike mutations detected after CCP usage.

## 4. Discussion

Escape from nAb-based therapeutics provides a crucial demonstration that these immune therapies target protective antigens, which the pathogen actively evades. Hence, the emergence of neutralizing-resistant variants in individuals receiving mAb and CCP provides powerful evidence for their antiviral activity. This evidence is independent of reduction in viral load, which has been reported with mAbs given early in disease but have been an inconsistent finding in randomized controlled trials (RCT) of CCP for COVID-19 [50].

Obtaining the frequencies for this phenomenon from case series is not possible due to the high risk of selection biases, which would yield unrealistically high frequencies. In contrast, RCTs with their control groups are the suggested reference. With bamlanivimab, resistance was reported in 7% of patients, regardless of dosage (700/2800/7000 mg) versus <1% in patients treated with placebo [22,29]. Apart from registration trials, the largest case series to date evaluated the impact of mAbs on the nasopharyngeal (NP) viral load and virus quasi-species of mAb-treated patients using single-molecule real-time sequencing after bamlanivimab alone (4 patients), bamlanivimab/etesevimab (23 patients) and casirivimab/Imdevimab (5 patients) [32]. To date a single case of immune escape has been reported for the non-overlapping REGN-COV2 cocktail, and accordingly hamster models and clinical trials showed no emergence of variants [51]. Since mAb therapy by definition targets only a single epitope within the RBD, it is unsurprising that escape mutations observed after in vitro and in vivo selection by these mAbs were single amino acid substitutions localized almost exclusively to the RBD (Figure 2, bottom panel; Table 1 and Table 2), as expected from in vitro studies with single mAb, but largely prevented by non-overlapping mAb cocktails [52].

In contrast to mAb therapeutics, immune escape under CCP has not been investigated in RCTs. Hence, evidence exclusively stems from case series and case reports [53] and is further complicated by exposure to multiple CCP units from different donors, each one having a polyclonal response at differing titers and affinity. Unfortunately, nAb titers were very rarely determined or reported, precluding correlation between the emergence of resistance and subneutralizing CCP doses. Overall, it seems that escape variants from CCP selection have not been reported as commonly nor emerged as fast, e.g., none of the eight recipients of hematopoietic stem cell transplantation or chimeric antigen receptor T (CART) lymphocytes who were treated with CCP and tested SARS-CoV-2-positive for 2 months showed significant mutations compared to the original strain [54]. A review of the spike protein changes associated with resistance after CCP therapy reveals that most of them had in-frame amino acid deletions in a flexible region that is partially solvent exposed and forms a β strand: plasticity may contribute to the structural permissibility of the identified deletions. The NTD is a flexible region that can be affected by immune escape via either insertions (causing additional glycosylation sites [12]) or recurrently deleted regions (RDR) ΔHV69–70 (RDR1), ΔLGVY141–144 and ΔD146 (RDR2), ΔI210 (RDR3) and ΔAL243–244 (RDR4) [55]: RDR1, RDR2 and RDR4 correspond to NTD loops N2, N3 and N5, whereas RDR3 falls between N4 and N5.

Deletions of amino acids from a protein structure generally result in greater structural changes than single amino acid changes, since these reduce the size of the protein and can trigger changes that propagate through the whole structure. Furthermore, the mechanism for the emergence of deletion variants appears to be very different from the single amino acid changes that are frequent from error-prone RNA replication and could involve deletions from RNA editing. Since CCP targets a large number of epitopes in the spike protein while mAbs target a single epitope, these molecular differences parallel what is expected from their respective selection pressures in the sense that escape from polyclonal preparations requires larger antigenic structural changes than escape from mAbs. In contrast to escape mutations selected for by mAb therapy, CCP selection yields point mutations throughout the spike protein. This reflects the vast antigenic surface area covered by the polyclonal antibodies within CCP. Escape mutations would be theoretically selected for on the basis of the most potent antibodies present in a particular CCP unit, which may vary markedly from donor to donor, which could explain the generally divergent evolution of SARS-CoV-2 in the presence of CCP. However, residues 141–144 and 243–244 are the sites of mutations or deletions in several cases, indicating these sites may offer effective escape from CCP derived from many donors, possibly by triggering a large-scale conformational rearrangement, as discussed above. As RBD binding antibodies are often neutralizing via ACE2 receptor occlusion, it is interesting that only 23% of CCP case studies identified the escape mutations within the RBD (Figure 2, top panel; Table 3). This suggests that antibody binding to other sites on the spike protein may have additional mechanisms of neutralization (i.e., by preventing conformational change after ACE2 engagement), or that additional antibody mediated immune responses (e.g., ADCC) are equally important as direct neutralization to the antiviral response to SARS-CoV-2.

Nothing can be inferred about the fitness of an emerging mutant in the absence of selective pressure, but it is of interest that one variant with the E484K mutant that emerged after bamlanivimab therapy was able to infect multiple household contacts [27]. In vitro, several mutants showed similar infectivity to the wild-type strain but resistance to different CCP donors [36]. In one instance of immune escape associated with CCP, a variant with D796H mutation manifested modestly reduced sensitivity to neutralization by CCP that was associated with reduced infectivity, which was only partly compensated by ΔHV69–70 [36]. Even if immune escape in registration trials has been a rare phenomenon, it should be considered that in real-world practice, mAbs targeting of the SARS-CoV-2 spike protein is being reserved for use in high-risk (immunocompromised) patients. Considering the huge size of the pandemic, the likelihood of immune escape becomes relevant, raising the possibility that rare variants with enhanced fitness could drive the next pandemic waves. Notably, several mutations have recurred in VOC and VOIs (e.g., E484K found in Beta and Gamma, E484Q found in Delta, or ΔLHR244–246 [41] found in VOI lambda), raising the possibility that such variants emerged during the treatment of patients (iatrogenic variants), but such inference will likely remain very hard to prove. E406W mutation, which causes resistance to REGN-COV-2, has never been reported in GISAID, and other E406 mutations remain exceedingly rare (worldwide, 318 cases of E406Q, 41 cases of E406D, and 2 cases each from USA for E406G, E406A, E406K, and 1 case of E406V out of 4,410,787 sequences deposited in GISAID as of 13 December 2021). The same is true for sotrovimab resistance, with E340 and P337 mutations exceedingly rare to date (E340K in 159 sequences worldwide, P337R in 18, P337L in 195, E340A in 105, E340G in 36, P337H in 44, P337T in 90) (source: Outbreak.info). Similarly, Q493R, which causes resistance to bamlanivimab + etesevimab, had only been reported in 244 sequences and Q493K in 138 sequences, before becoming one of the hallmark mutations of VOC Omicron. L452R, which causes resistance to regdanvimab, also became prevalent first in VOI Epsilon and then in VOC Delta (source: Outbreak.info). Lack of fixation of those mutations facilitates the imputation that these require mAb selective pressure and/or effective infection control techniques in the care of those patients to prevent spill over to the general population.

Within-host variation (so-called “quasi-species swarm”) is a natural phenomenon which has been reported for SARS-CoV-2 in immunocompetent patients and ultimately facilitates the persistence of infection. Among 33 patients having positive NPS PCR for an average of 18 days, Voloch et al., observed a distinguishing pattern of mutations over the course of the infection mainly driven by increasing A→U and decreasing G→A signatures, including spike mutations (V362L, T553I, H655Y, A688V, S691F, S884F, V1176F). G→A mutations are driven by the RNA-editing enzyme activities typical of innate immunity [56]. Nevertheless, several covariates can facilitate immune escape.

Immunosuppression has been postulated to be an accelerator for viral evolution. Actually, Table 4 shows that very few case reports have detailed intraclonal (within-host) evolution in patients receiving immunosuppressive treatment, and, in the absence of nAb-based therapeutics, spike mutations rarely occurred [54].

On the other hand, co-administered small chemical antivirals can be mutagenic per se. Remdesivir has both amino and imino tautomers when pairing with RNA bases [57]. Both amino-remdesivir:G and imino-remdesivir:C pairs are mutagenic. It has hence been been proposed than nAb-based therapeutics could amplify the mutations induced by remdesivir [3]. In this regard, Table 4 shows that many of the mAb- or CCP-associated mutations emerged in individuals who were or had been treated with remdesivir (but neither mAbs nor CCP), consistent with the notion that antiviral therapy could potentiate the emergence of antibody-resistant mutations.

## 5. Conclusions

In summary, our survey of the available mutational data show that escape variants associated with mAb and CCP therapy manifest different type of mutations. For mAbs, most mutations are single amino acid replacements in the RBD domain, while most variants eliciited in patients treated with CCP exhibited amino acid deletions. In fact, it is noteworthy that RBD mutations were relatively rare in CCP escape variants. Although the numbers are relatively small, which suggests caution in making generalizations, this dichotomy in geography of mAb and CCP mutations could reflect the fact that mAbs target a single epitope where the mAb–antigen interaction can be significantly altered by single amino acid changes while CCP targets many epitopes and has several mechanisms of action, such that evading polyclonal antibody immunity is likely to require much larger spike protein structural changes. Despite the relatively small set of variants for which there is molecular data available, the large variation of molecular solutions that allow SARS-CoV-2 to escape antibody-mediated protection is striking and suggest the need for continued vigilance in genomic surveillance, especially in cases refractory to therapy.

## Figures and Tables

**Table 1:** Spike mutations associated with clinically-approved mAb resistance in vitro, sourced via the Stanford University Coronavirus Antiviral and Resistance Database (accessed online on 13 December 2021, at https://covdb.stanford.edu/search-drdb/). Mutations conferring resistance to both mAbs within the cocktail are underlined.

Manufacturer	Cocktail Brand Name	Active Ingredient (Brand Name)	Spike Mutations Associated with In Vitro Resistance	Ref
Eli Lilly (AbCellera/Junshi)	n.a.	etesevimab (LyCoV016, CB6, JS016, LY3832479)	K417N/T (100 folds), D420N (100 folds)F456R/A/K (100 folds) N460K/S/T/Y (50–100 folds) I, I472D A475R/V (20–100 folds), E484K N487R (100 folds), G485P, Q493R/K (100 folds)	[9,10,19]
		bamlanivimab (LY-CoV555, LY3819253)	L452R (>100 folds,) E484D/K/Q (>100 folds) G485P, F490S/L (100 folds) Q493R/K (100 folds) S494P/R (100 folds)	
Regeneron/Roche	REGN-COV2 (Ronapreve)	imdevimab (REGN10987)	E406W (>100 folds) N439K (25–100 folds) N440K (28–96 folds) K444L/M/N/Q/T (>100 folds), V445A (>100 folds), G446V (>100 folds) N450D (9–32 folds) Q498H (17 folds) P499S (>100 folds) E484K (16 folds)	[9]
		casirivimab (REGN10933)	E406W/D (50–93 folds) K417E/N/R/T (25–100 folds) V455T (>100 folds) Y453F (>100 folds) L455F (80 folds) A475R (44 folds) E484K/Q (20–55 folds), F486x F486K/L/R/S/V (>100 folds) N487R (>100 folds) Q493E/K/R (25–100 folds)	
AstraZeneca	AZD7442 long-acting antibody (LAAB)(Evusheld)	tixagevimab (AZD8895/COV2-2196)	E484K (4–11 folds) S982A (3.2 folds)	[13,20,21,22]
		cilgavimab (AZD1061/COV2-2130)	E484K (3.2 folds)	
Celltrion	-	regdanvimab (CT-P59) (Regkirona)	L452R (35 folds) E484K (8.7 folds) N501Y (5.5 folds)	[23]
GSK	-	sotrovimab (VIR-7831, S309, GSK4182136) (Xevudy)	P337R/L/H/T (180–276 folds) E340K/A/G (27–300 folds)	[24,25]

**Table 2:** Case reports of immune escape after anti-spike mAb treatment.

mAb Type	Age/Sex (Identifier)	Condition (Treatments)	Day mAb Adminstered	Other Antiviral Treatments	SARS-CoV-2 Strain	Spike Mutations	First Detected at Day	Outcome	Ref
REGN-CoV2 cocktail	45/M	antiphospholipid syndrome (steroids, rituximab, ruxolitinib, IVIg, cyclophosphamide)	45	remdesivir (5 + 10 + 5 days)	n.a.	E484K/A, Y489H, Q493K and N501Y	75	death	Choi et al. [20] Clarke et al. [21]
bamlanivimab monotherapy (700 mg iv)	n.a./n.a. (B2_11)	immunocompetent	median 4.5	n.a.	n.a.	E484K	3 after mAb	n.a.	Choudhary et al. [22]
	n.a./n.a. (B2_10)			n.a.	n.a.	S494P	6 after mAb	n.a.	
	n.a./n.a. (B2_8)			n.a.	n.a.	E484K	4 after mAb	n.a.	
	n.a./n.a. (B2_7)			n.a.	n.a.	E484K	6 after mAb	n.a.	
	n.a./n.a. (B2_6)			n.a.	n.a.	S494P + E484K (frequency < 20%)	3 after mAb	n.a.	
	n.a./n.a. (B2_5)			n.a.	n.a.	E484Q	4 after mAb	n.a.	
	n.a./n.a. (B2_4)			n.a.	n.a.	E484K	8 after mAb	n.a.	
	n.a./n.a. (B2_3)			n.a.	n.a.	S494P	6 after mAb	n.a.	
	n.a./n.a. (B2_2)			n.a.	n.a.	E484Q	3 after mAb	n.a.	
	72/M	chronic lymphocytic leukemia and hypogammaglobulinemia (venetoclax and rituximab 17 days earlier); steroids 21–26	4 (700 mg)	1 BNT162b2 dose 20 days before CPP day 10	Alpha	E484K and Q493R	6	recovered at day 61	Truffot et al. [23]
	55/F	acute myeloid leukemia	14	Remdesivir days 23–27	Alpha	E484K and Q493R, S494P	21	negative at day 51	Lohr et al. [24]
	70/M	ANCA-associated vasculitis with end-stage renal disease (rituximab and prednisolone)	2	3 units of CCP at day 16	B.1	E484K → E484Q, reverted to E484K after CCP	12	died of MOF on day 20	Jensen et al. [25]
	40/F	AIDS	3	remdesivir and 2 units of CCP	B..1.	E484K	10	recovered	
	60/M	relapsed follicular lymphoma (obinutuzumab, thiotepa, cytarabine, etoposide)	76	2 CCP units on day 57 and 1 CCP unit on day 59	B.1.177	E484K	87	recovered, negative at day 103	
	65/M	heart transplant recipient (about 30 years ago) (cyclosporine, azathioprine, prednisolone)	2	none	B.1.177	E484K	19	discharged at day 40 after 2 negative NPS	
	65/M	chronic lymphatic leukemia	45	remdesivir and 3 units of CCP days 52-62, imdevimab/casirivimab about day 70	B.1.258	E484K	52	recovered, negative at day 91	
	33/M	Hodgkin lymphoma (untreated)	20	dexamethasone	B.1.362	ΔF140 → ΔPFLGVY139–144, G485R, W258C	45	hospitalized for HL chemotherapy at end of follow-up	Bronstein et al. [26]
	68/M	chronic lymphocytic leukemia (FCR in 2017, prednisone for AIHA, venetoclax + rituximab in 2019)	10	CPP days 12 and 26, IVIg day 21, remdesivir days 37-41	Alpha	E484Q	22	discharged day 43	
	n.a.	immunocompetent	2	n.a.	B.1.311	E484K	n.a.	resolved at home	Sabin et al. [27]
	87/M	immunocompetent	2	none	Alpha	E484K +S494P	6	discharged negative at day 27	Peiffer-Smadja et al. [28]
	35/M	immunocompetent	2	none	Alpha	E484A/K	6	discharged, negative at day 38	
	61/M	immunocompetent	2	steroids	Alpha	E484K	12	negative at day 8, hospitalized for unrelated reasons	
	97/M	immunocompetent	4	none	Alpha	E484K	14	died at day 35 because of soft tissue infection	
	64/M	heart transplant recipient	2	corticosteroids for 10 days	Alpha	Q493R	26	discharged, negative at day 48	
bamlanivimab 700 mg + etesevimab 1400 mg cocktail	n.a.	n.a.	2	n.a.	n.a.	S494P	11	recovered, not detected in samples at day	Gottlieb et al. [29]
	73/M	cholangiocarcinoma (steroids)	2	none	Alpha	Q493R	7	died day 18	Focosi et al. [30]
	63/M	allogeneic hematopoietic stem cell transplantation recipient for mycosis fungoides	n.a.	none	Alpha	Q493R	15	discharged at day 2	Guigon et al. [31]
	n.a.	solid organ transplantation	n.a.	none	Alpha	Q493R	7	n.a	Vellas et al. [32]
	n.a.	solid organ transplantation	n.a.	none	Alpha	Q493R	7	n.a.	
	n.a.	solid organ transplantation	n.a.	none	Alpha	Q493R	14	n.a.	
	n.a.	solid organ transplantation	n.a.	none	Alpha	Q493K	7	n.a.	
	n.a.	solid organ transplantation	n.a.	none	Alpha	E484K	21	n.a.	
	34/F	B-ALL	<5 days	2 CCP units days 29–30	Alpha	Q493R	n.a.	all were rescued with CCP	Pommeret et al. [33]
	62/F	Hodgkin lymphoma		1 CCP unit day 26	Alpha	Q493R	n.a.		
	63/F	follicular lymphoma		2 CCP units days 30–31	Alpha	Q493R	n.a.		
	67/F	follicular lymphoma		2 CCP units days 15–16	Alpha	n.a.	n.a.		
	57/M	chronic lymphocytic leukemia		2 CCP units days 30–31	Alpha	E484D	n.a.		

**Table 3:** Case reports of immune escape after CCP treatment.

Age/Sex (Identifier)		Condition	CCP Schedule (and Titer)	Co-Treatments	SARS-CoV-2 Strain	Spike Mutations	First Detected at Day	Outcome		Ref
71/F		chronic lymphocytic leukemia and iatrogenic hypogammaglobulinemia	70 (1:60) and 81 (1:160)	IVIG q4–6w	n.a.	ΔPFLGVYY139–145	49	negative NPS since day 105		Avanzato et al. [34]
						ΔLGVY141–144	70 (poor causality)			
73/M		chimeric antigen receptor T-cell recipient	low titer days 2 and 58	remdesivir days 5–10, 63–74 dexamethasone days	GH	R190K and G1124D	13	died day 74		Hensley et al. [35]
						ΔY144, D215G, and N501T	67			
						ΔH146	72			
70/M		B-cell depletion and hypogammaglobulinemia	63, 65, 102	remdesivir day 38–48, 52–62 and 91–101	n.a.	D796H and ΔHV69–70	57	died on day 102		Kemp et al. [36]
21/M		B-acute lymphoblastic leukemia (tisagenlecleucel)	78, 103, 110, 123, 130, 137, 144, 158, 165, 172	remdesivir (2 × 5–day courses)	n.a.	3 major allele variants emerged between days 0 and 40 with an additional 4 major and 7 minor allele variants by day 144 (ΔLGV141–143, ΔY145, ΔLGVY141–144, ΔNL211–212, N440K, V483A, and E484Q)	144	positive NPS at end of follow-up (day 250)		Truong et al. [37]
50/M		kidney transplant recipient (tacrolimus, steroids)	1	tocilizumab day 2	B.1.369	Q493R, ΔAL243–244 had ~70% frequency; ΔLGVY141–144, E484K and Q493K had ~30%, ~20% and ~10% frequency	21	died on day 94		Chen et al. [38]
75/M		B-CLL (FCR, ibrutinib)	2 units on day 70, 2 units on days 127–128	remdesivir days 24–33 and 60–64	n.a.	H49Y, ΔY144, ΔLLA241–243, ΔAL243–244, L242H, A243P, F490S, N1178N, and C1250F	80	still positive at end of follow-up (day 333)		Monrad et al. [39]
60/M		mantle-cell lymphoma and associated B-cell immunodeficiency (rituximab, bispecific mAb, cyclophosphamide, doxorubicin, prednisone)	31, 122	remdesivir day 30 and 122	n.a.	mutations in ORF1a but not in spike	n.a.	still positive at end of follow-up (day 156)		Baang et al. [40]
40/F		diffuse large B-cell lymphoma (chimeric antigen receptor T lymphocytes) and hypogammaglobulinemia	high-titer day 2, 313	IVIG, remdesivir day 2 and 313	B.1.332	ΔLHR244–246 and A243G	313 (poor causation)	discharged day 324, cleared at day 335		Nussenblatt et al. [41]
70/F (A)		follicular lymphoma (obinutuzumab-CHOP)	23, 34, 49, 55, 56, 62, 65, 70, 73, 77, 84, 86, 90, 94, 106	steroids	B.1.1.29	L18F, R682Q, ΔY144	50	died 5 months later		Khatamzas et al. [42]
70/M		mantle cell lymphoma (R-BAC)	88	darunavir/ritonavir, hydroxychloroquine, methylprednisolone, tocilizumab days 1 78, remdesivir days 45–50 and 78–87, 180–184 and 210–214, IVIg	B.1.1	H69Y/P, V70G and S982A	238	died on day 271, still positive at day 268		Sepulcri et al. [43]
40/M		autologous hematopoietic stem cell transplant due to a diffuse large B-cell lymphoma	2 doses on days?	IVIg	B.1.128	ΔLGV141–143 → ΔLGVY141–144	134	negative PCR on day 196		Mendes-Correa et al. [44]

**Table 4:** Intrahost variation in spike sequence detected in immunocompromised patients not receiving nAb-based treatments.

Age/Sex (Identifier)	Condition	Antiviral Treatments	SARS-CoV-2 Strain	Spike Mutations	First Detected at Day	Outcome	Ref
47/F	diffuse large B cell lymphoma (rituximab plus polychemotherapy)	n.a.	B.1.1.163	Y453F, ΔHV69–70, S50L, ΔLGVY141–144, T470N, and D737G	120	negative PCR on day 132	Bazykin et al. [7]
61/F	diffuse large B cell lymphoma stage IVB	remdesivir for 10 days, high-dose steroids for 7 days	B.1.1.401	V3G, S50L, N87S, A222V, ΔLTTRTQLPPAYTN18–30 and ΔLGVY141–144	164	negative PCR at day 197	Borges et al. [45]
3/F (1)	B-cell acute lymphoblastic leukemia (chemotherapy)	n.a.	20C	silent I410I (22792:C/A)	27	negative PCR at day 91	Truong et al. [37]
2/M (3)	B-cell acute lymphoblastic leukemia	remdesivir for 5 days	20C	V483A and E484Q	139	negative PCR at day 196	
				V70P, ΔLGV141–143, N440K	162		
37/F	advanced HIV and antiretroviral treatment failure	dexamethasone	B.1.1.273	E484K	6	negative at day 233	Karim et al. [46]
				K417T and F490S	71		
				L455F and F456L	106		
				D427Y and N501Y	190		
80/M	chronic lymphocytic leukemia and hypogammaglobulinemic	remdesivir days 213–230, REGN-COV-2 day 265	B.52	L179	58	negative PCR day 311	Kavanagh Williamson et al. [47]
				S255F, S477N, H655Y, D1620A, ΔHV69–70	155		
40/M	autologous hematopoietic stem cell transplant due to a diffuse large B-cell lymphoma	IVIg	B.1.128	ΔLGV141–143 → ΔLGVY141–144		negative PCR on day 196	Mendes-Correa et al. [44]
n.a./n.a.	transplant recipient	remdesivir	n.a.	S13I, T95I, E484G, F490L, ΔLGVY141–144, ΔLHRS244–247, and ΔSPRRARSV680–687	n.a.	n.a.	Weigang et al. [48]
n.a./n.a.	18 B-cell non-Hodgkin lymphoma	44% CCP 37% remdesivir	n.a.	n.a.	requested	n.a.	Lee et al. [49]

## Data Availability

The data presented in this study are openly available in PubMed, medRxiv and bioRxiv.

## Abbreviations

nAb	neutralizing antibodies;
CCP	COVID-19-convalescent plasma;
PSM	propensity score-matched
RCT	randomized controlled trials
